# Involvement of Wnt/β-catenin signaling in the mesenchymal stem cells promote metastatic growth and chemoresistance of cholangiocarcinoma

**DOI:** 10.18632/oncotarget.5514

**Published:** 2015-10-14

**Authors:** Weiwei Wang, Wei Zhong, Jiahui Yuan, Congcong Yan, Shaoping Hu, Yinping Tong, Yubin Mao, Tianhui Hu, Bing Zhang, Gang Song

**Affiliations:** ^1^ Cancer Research Center, Medical College of Xiamen University, Xiamen 361102, China; ^2^ Department of Basic Medicine, Medical College of Xiamen University, Xiamen 361102, China

**Keywords:** cholangiocarcinoma, MSCs, metastasis, chemoresistance, β-catenin

## Abstract

Mesenchymal stem cells (MSCs) are multi-potent progenitor cells with ability to differentiate into multiple lineages, including bone, cartilage, fat, and muscles. Recent research indicates that MSCs can be efficiently recruited to tumor sites, modulating tumor growth and metastasis. However, the underlying molecular mechanisms are not fully understood. Here, we first demonstrated that human umbilical cord-derived mesenchymal stem cells (hUC-MSCs), when mixed with human cholangiocarcinoma cell lines QBC939 in a xenograft tumor model, significantly increased the cancer cells proliferation and metastatic potency. MSCs and their conditioned media (MSC-CM) could improve the drug resistance of tumor when the compound K (CK) as an anti-cancer drug, a major intestinal bacterial metabolite of panaxoside, was administered to xenograft tumor mice. Furthermore, MSCs greatly increased the colony formation and invasion of cholangiocarcinoma cells QBC939 and Mz-ChA-1. Immunochemistry studies of cholangiocarcinoma tissue chips and transplantation tumor from nude mice showed that the expression of β-catenin was important for cholangiocarcinoma development. We further demonstrated that MSCs and MSCs-CM could promote proliferation and migration of cholangiocarcinoma cells through targeting the Wnt/β-catenin signaling pathway. hUC-MSCs or MSCs-CM stimulated Wnt activity by promoting the nuclear translocation of β-catenin, and up-regulated Wnt target genes MMPs family, cyclin D1 and *c*-Myc. Together, our studies highlight a critical role for MSCs on cancer metastasis and indicate MSCs promote metastatic growth and chemoresistance of cholangiocarcinoma cells *via* activation of Wnt/β-catenin signaling.

## INTRODUCTION

Cholangiocarcinoma is a malignancy originating from the bile ducts with features of cholangiocyte differentiation. Based on anatomical location, it can be classified into intrahepatic, perihilar, and distal cholangiocarcinoma [1]. It is the second most common primary hepatic malignancy, after hepatocellular cancer, and epidemiologic studies indicate that its incidence is increasing worldwide [2, 3]. Cholangiocarcinoma is commonly diagnosed in the advanced stage of the disease and has a dismal prognosis. Moreover, cholangiocarcinoma can easily metastasize and cause relapse. Surgical treatment is the preferred option for all types of cholangiocarcinoma, but many factors, such as the vascular structures and lymph nodes, needs to be considered [4, 5]. The recurrence and metastasis in cholangiocarcinoma has become a key point which affecting the efficiency and obtaining long-term survival in patients. Understanding of the cancer biology, the mechanism of the metastasis, and its complex interaction with the tumor microenvironment could lead to optimum therapies with improvement in patient survival. In recent years, a growing number of studies have reported mesenchymal stem cells (MSCs) have a close relationship with tumor proliferation and metastasis. Study of MSCs and tumor microenvironment has also become a hot spot of cancer research.

MSCs are non-hematopoietic stem cells, which are capable of differentiation at least into bone, cartilage, muscle, and adipose tissues [6]. They reside primarily in the bone marrow, and have the ability to recruit to their destination in response to systemic signals emanating from injured tissues, inflammatory sites or primary tumor sites [7–9]. Many reports described that MSCs can home and engraft to different types of solid tumors, such as breast [10, 11], prostate [12], lung [13], and ovarian [14]. In addition, MSCs are relatively nonimmunogenic and have the ability to expand manifold in culture without lose its multilineage potential. All these characters make MSCs extremely attractive for targeted cancer therapy. On one hand, MSCs may affect cancer progression through secreted factors triggering activation of various cell signaling pathways. On the other hand, they can be used as cellular vehicles for cancer-targeted gene therapy [15].

MSCs and their secreted extracellular proteins are crucial for establishing the tumor microenvironment. Although the interactions of MSCs and many cancers have been widely studied, the functional mechanisms of MSCs on cholangiocarcinoma progression are poorly understood.

In the tumor microenvironment, upon with MSCs, cancer cells may exhibit altered biological functions, including proliferation ability, migration and drug resistance. Drug resistance is one of the major obstacle in cancer treatment. The mechanism of cancer cell drug resistance include enhanced activity of positive regulators of cell proliferation, inactivation of cell death or enhancement of survival functions and activation of telomerase [16]. Chen et al. demonstrated that MSC-secreted CM could induce doxorubicin resistance in TNBC, which was mediated by IL-8 presented in the MSC-CM [17]. MSCs also exhibited increased chemosensitivity and induction of apoptosis in response to doxorubicin and 5-fluorouracil, reported by Lucia group [18]. Xia et al. concluded that MSCs protected leukemia cells from apoptosis, at least in part, through *c*-Myc dependent mechanisms, but cell-cell contact is required [19]. Considering the important role of MSCs on cancer cell drug resistance, we extended our investigation on the effect of stromal cells on drug responses in the tumor cells, using compound K as an effective anti-cancer drug. compound K is one of the major intestinal bacterial metabolite of protopanaxadiol-type saponins formed from ginsenosides Rb1, Rb2, and Rc. Compound K has received increasing attention because of its various pharmacological activities including anti-tumor, anti-inflammatory, and anti-diabetic effects [20–22].

Here, using multiple *in vitro* and *in vivo* models, we examined the roles of hUC-MSCs in the progression of cholangiocarcinoma development, and revealed the cellular and molecular mechanisms by which MSCs promote cholangiocarcinoma development. Our study first demonstrated that MSCs or their CM significantly increased cholangiocarcinoma cells proliferation, metastatic potency and chemoresistance both *in vitro* and *in vivo*. Furthermore, we found that metastasis of cholangiocarcinoma is associated with the translocation of β-catenin and upregulation of cyclinD1, *c*-Myc and MMP2. Such findings indicated that MSCs played a promoting role in cholangiocarcinoma cells progression and metastasis.

## RESULTS

### MSCs promote cholangiocarcinoma cells proliferation and tumorigenesis

hUC-MSCs (hereafter referred to as MSCs) isolated from Wharton's jelly of umbilical cord and characterized by flow cytometry with antibodies to cell surface markers. Microscopy showed that cells with spindle shape spread on cell culture plate, and the cells were positive for CD29, CD44, CD90, and negative for CD34, CD45 (Supplementary Figure S1A and S1B). In addition, their ability to differentiate into fat and bone were tested and verified (Supplementary Figure S1C, S1D).

**Figure 1:**
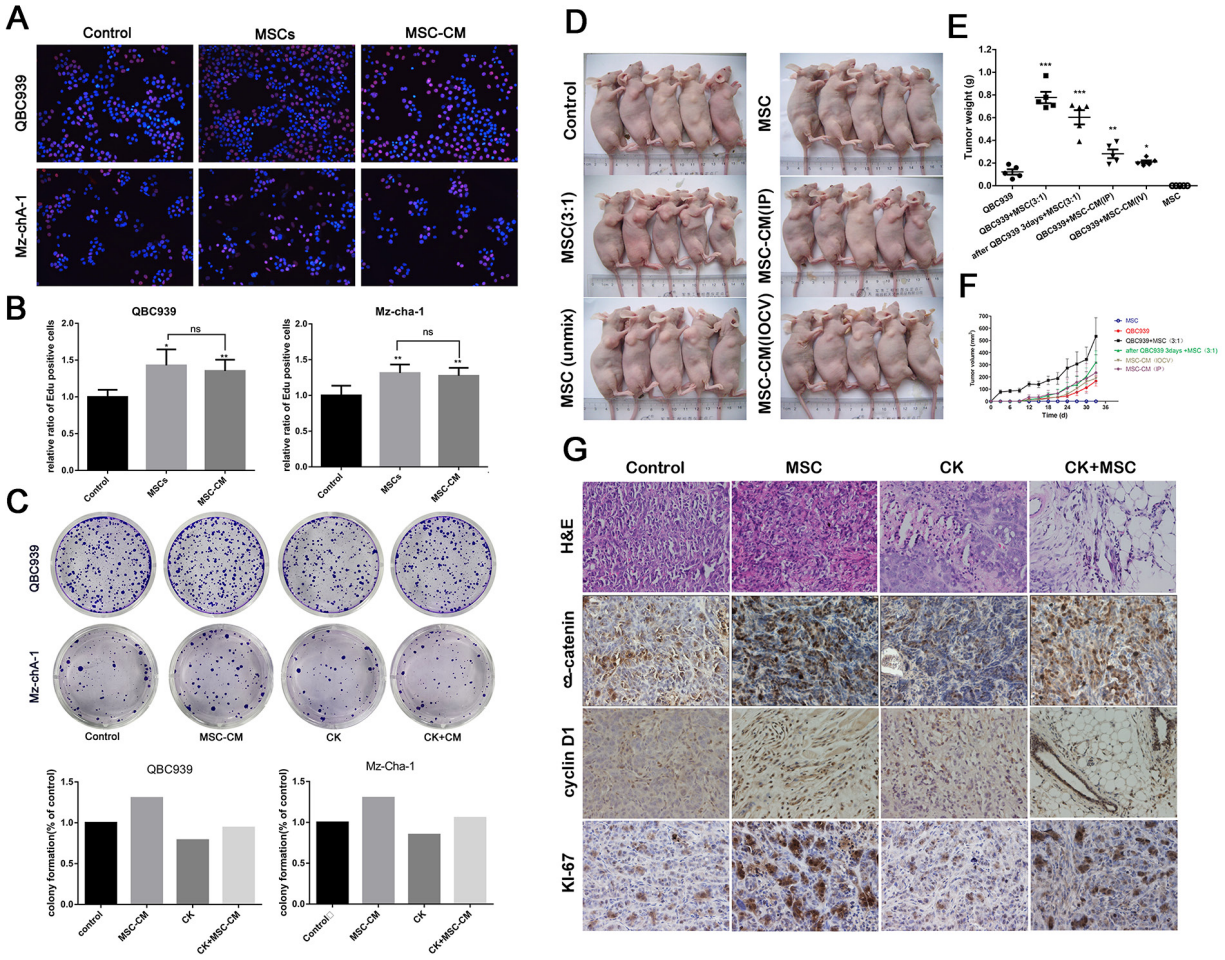
The promotion effects of MSCs on cholangiocarcinoma **A and B.** The Edu proliferation assay was performed 24 hours after QBC939 and Mz-chA-1 cells treated with MSCs or MSC-CM. A: representative image; B: ratio of EdU-positive cancer cells. **C.** Representative images of colony-forming assay and its counting results. **D.** Nude mice were implanted with cell alone or mixed-cell and treated with MSC-CM or RPMI 1640 via different ways. *N* = 5 mice per group; **E and F.** the statistical results of tumor volume and weight. **G.** The H&E staining and Immunohistochemical analysis about expression of β-catenin, cyclin D1, Ki-67 in tumor tissue. Data are reported as means ± S.D. of three separate experiments. * and ** indicate *p* < 0.05 and *p* < 0.01 compared with control group, respectively. Abbreviations: MSC-CM, mesenchymal stem cell conditioned medium; CK, compound K; H&E, hematoxylin-eosin staining.

In order to determine whether MSCs exhibit tumor supportive or inhibitory effect on cholangiocarcinoma cells, we performed a co-culture experiment. QBC939 and Mz-ChA-1 cells were mixed with MSCs cells or MSC-CM produced from corresponding amount of MSCs. 24 hours later, proliferation ability was tested by Edu assay. The MSCs-induced promotion effects on cancer cell proliferation were significantly greater than in controls. The conditioned medium from MSCs also showed a remarkable effect (Figure 1A, 1B). To investigate whether MSCs enhances the colony-forming ability of QBC939 and Mz-ChA-1 cells *in vitro*, we seeded 500 cells in triplicate wells of six-well plates for a colony-forming assay. After cell adherence, cells were cultured with CK (5 μM) or MSC-CM for 12 hours. 2 weeks later, cells treated with MSC-CM formed clones were more numerous and larger than in control groups (Figure 1C). CK could inhibit the cholangiocarcinoma cells tumorigenesis, while MSC-CM have the ability to relieve the inhibiting effects of CK. Next, in order to determine the effect of MSCs on tumor growth *in vivo*, we established a xenograft model which QBC939 cells were mixed with MSCs and injected subcutaneously into immunocompromised mice. The growth kinetics of the MSC-containing tumors were compared to those of QBC939 injected alone over the 1–5 weeks. Besides, the results of the tumor weight, histopathology of the tumors were studied. As shown in Figure 1D, 1E, and 1F, The tumor mean volume and weight of the mixed-cell group and MSCs-treated group are dramatically higher than the control group. The mixed-cell group showed the highest record among all the groups. The expression of Ki67 in cancer tissues was shown in Figure 1G. The Ki67-positive cells exhibited brown punctate granules in the nucleus. We can see a higher expression of Ki-67 when QBC939 mixed with MSCs or MSC-CM. These results revealed that MSCs and MSC-CM promoted cholangiocarcinoma growth and tumorigenesis *in vitro* and *in vivo*.

### MSCs significantly increased the metastasis of cholangiocarcinoma

**Figure 2:**
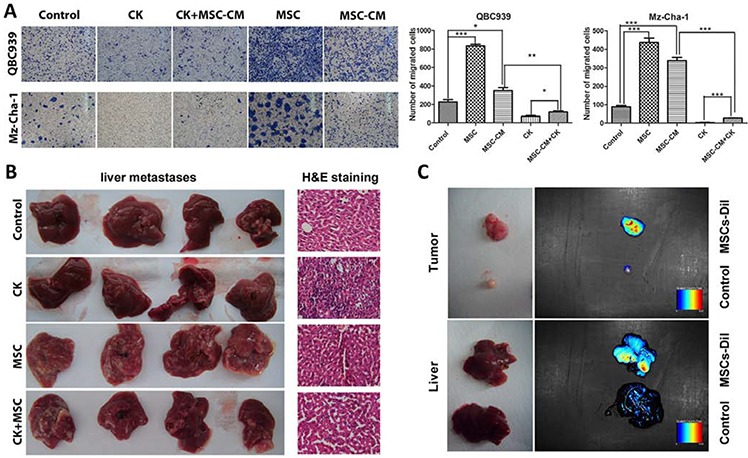
MSCs promote cholangiocarcinoma cancer metastasis **A.** QBC939 cells and Mz-ChA-1 cells were planted into up chamber of transwell with serum-free medium, MSC-CM or MSCs were in the down chamber, after 48 hours, the cells migrated to the bottom chamber were visualized by staining. Quantification is shown at right. **B.** Liver migration in different groups, and the H&E staining of each group. **C.** MSCs recruited to the tumor sites and metastatic livers of the tumor bearing nude mice. Data are reported as means ± S.D. of three separate experiments, * and ** indicate *p* < 0.05 and *p* < 0.01 compared with control group, respectively. Abbreviations: MSCs, mesenchymal stem cell; MSC-CM, mesenchymal stem cells conditioned medium; CK, compound K.

We next investigated the effects of the MSCs on *in vitro* invasion ability of cholangiocarcinoma cells and *in vivo* metastasis. We initially performed migration *assay* using *co-culture*, which the MSCs were seeded into the bottom of a plate, and cholangiocarcinoma cells were seeded into the up chamber. The results show that the effect of the MSCs on QBC939 cell migration was significantly higher than control cells. The MSC-CM also significantly promoted the migration of the cholangiocarcinoma cells, suggesting that soluble factors secreted by the MSCs were responsible for the effects. The QBC939 cells were replaced by Mz-ChA-1 cells to repeat the experiments mentioned above, similar results were obtained (Figure 2A).

From *in vivo* study, mice bearing the mixed QBC939+MSCs tumors display a marked increase in the number of macroscopic liver metastases (Figure 2B). Recent studies described that MSCs can recruited to many types of malignancy, such as gliomas, colon carcinomas, melanomas and breast carcinomas [10, 23–25]. We infused MSCs (labelled with CM-Dil) into the venous circulation of mice bearing QBC939 or QBC939/MSCs cells. As shown in Figure 2C, MSCs localized to the developing tumors, and even to the metastatic liver.

Such findings indicated that MSCs could be recruited by subcutaneous cholangiocarcinoma xenografts, and the metastasis-promoting ability were a specific property of admixed MSCs.

### MSCs greatly increased cholangiocarcinoma cell chemoresistance induced by compound K

**Figure 3:**
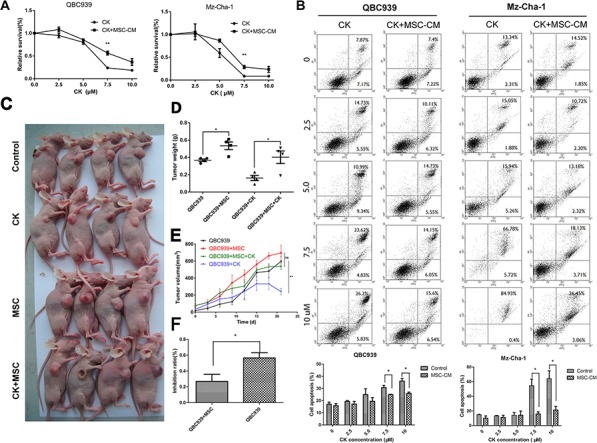
MSCs decrease the effects of Compound K on cholangiocarcinoma **A.** QBC939 and Mz-ChA-1 cells were treated with different concentration of CK (0, 2.5, 5.0, 7.5, 10 μM), which were dissolved with MSC-CM or serum-free medium for 24 hours. Cells were analyzed with MTS assy. Data are reported as means ± S.D. of three replicates. **B.** QBC939 and Mz-ChA-1 cells were treated with different concentration of CK (0, 2.5, 5.0, 7.5, 10 μM), at the same time, cells were treated with MSC-CM or serum-free medium for 24 hours and analysis with Flow Cytometry. Statistical results are shown below. **C.** QBC939 cells were implanted into nude mice alone or mixed with MSCs. After tumor emerges, mice were treated with CK (10 mg/kg) every other day. **D–F.** Tumor volume, weight and CK inhibit ratio were analyzed (*N* = 4 mice of each group). Data are reported as means ± S.D.* and ** indicate *p* < 0.05 and *p* < 0.01, compared with control group, respectively. Abbreviations: CK, compound K; MTS, 3-(4,5-dimethylthiazol-2-yl)-5-(3-carboxymethoxyphenyl)-2-(4-sulfophenyl)-2H-tetrazolium.

CK, a ginsenoside metabolite, has been shown to inhibit proliferation and induces apoptosis in a variety of cancers by modulation of diverse signal pathways [20]. Since there has been limited evidence that CK could suppress cholangiocarcinoma cell growth, we performed experiments using QBC939 and Mz-ChA-1 cells *in vitro* and *in vivo*. Firstly, from the colony formation assay, an apparent decrease of cell-colony formation was observed in QBC939 and Mz-ChA-1 cells treated with CK (Figure 1C). For MTS assay, an inhibition of cell viability was observed in two cholangiocarcinoma cell lines after being treated with different concentration of CK. While treated with CK along with MSCs-CM, cholangiocarcinoma cells viability partly elevated, and CK-induced cell death cells decreased (Figure 3A). Previously, our studies showed that CK can induce apoptosis in liver cancer cells [22]. To determine whether the proliferation inhibitory effect of CK on QBC939 and Mz-ChA-1 cells was also associated with the process of apoptosis, and the effects of MSCs on cancer cell apoptosis, we did an annexin V/PI apoptosis assay. After exposure to CK for 24 hours, the proportion of apoptotic cells increased. However, MSCs conditioned medium significantly inhibited QBC939 and Mz-chA-1 cell apoptosis compared to the CK group, especially CK concentration was 7.5 μM or 10 μM (Figure 3B).

Furthermore, we investigated the effect of CK and MSC-CM on the metastases of cholangiocarcinoma *in vivo*. QBC939 and QBC939 mixed with MSCs were injected subcutaneously into immunocompromised mice. CK was injected by enterocoelia every other day at a concentration of 10 mg/kg. Three weeks later, mice were sacrificed (Figure 3C). From Figure 3D and 3E we can concluded that MSCs promote QBC939 growth in nude mice, whereas CK inhibit QBC939 growth. What's more, compared the inhibit ratio about MSCs group with control group, we found that in MSCs group CK inhibit ratio is lower than the control group (*p* < 0.05) (Figure 3F). These result revealed that MSCs and their conditioned medium could decrease the susceptibility of cancer cells to CK.

### MSCs increased β-catenin expression and activated Wnt signaling

Accumulated evidence proved that Wnt signaling pathway played an important role in cancer cell progression, including proliferation and metastasis [28, 29]. Aberrant activation of the Wnt signaling pathway may lead to malignancy [30]. So we examined whether cholangiocarcinoma progression was associated with Wnt signaling. We used a tissue chip which contains 42 cholangiocarcinoma tissues to detect the expression of β-catenin and *c*-Myc (Supplementary Figure S2). Immunohistochemistry results showed that β-catenin was related to Pathologic Tumor-Node-Metastasis (PTNM) of cholangiocarcinoma (Tables 1, 2), which indicated that β-catenin may play an important role on cholangiocarcinoma development. Meanwhile, it provided us a possible evidence to study the mechanism of MSCs effect on cholangiocarcinoma.

**Table 1:** β-catenin and *c*-Myc expression in cholangiocarcinoma and para-carcinoma tissues

organization pattern	numbers	β-catenin		positive (%)	*c*-Myc		positive (%)
		−	+		−	+	
carcinoma tissue	42	9	33	78.6	25	17	40.5
para-carcinoma tissue	42	10	32	76.2	12	30	71.4

**Table 2:** β-catenin expression in different cholangiocarcinoma tissues

pathological factors	Numbers	β-catenin		positive (%)	X^2^	*p*
		−	+			
**sex**						
male	21	6	15	71.4	1.2727	>0.05
female	21	3	18	85.7		
**age**						
≤60	27	5	25	83.3	1.4141	>0.05
>60	15	4	8	66.7		
**PTNM**						
I	3	2	1	33.3	3.9269	<0.05
II + III	39	7	32	82.1		

**Figure 4:**
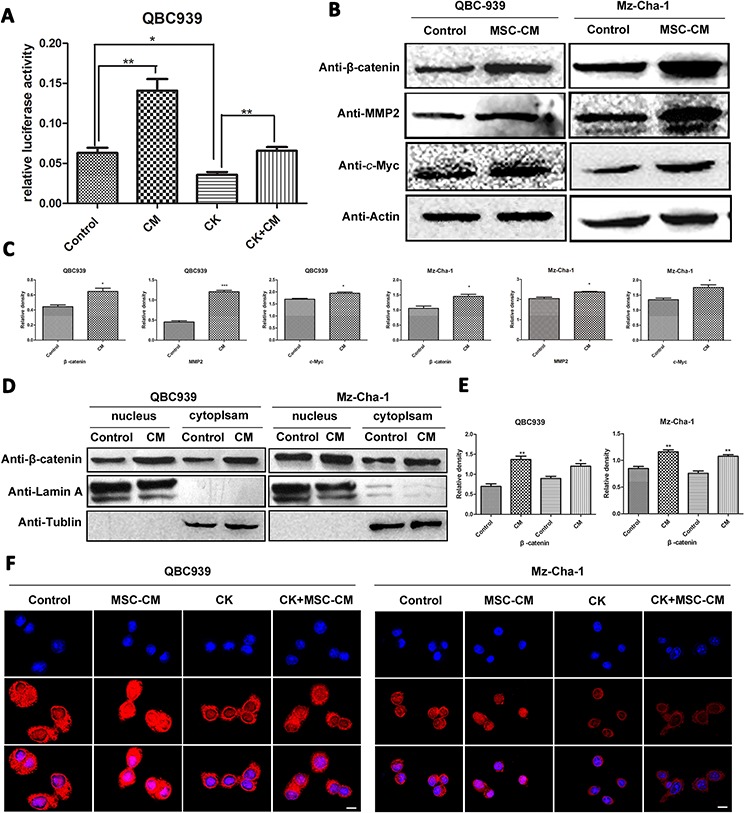
Effects of MSCs-CM on Wnt-related proteins in human cholangiocarcinoma cells **A.** Effect of MSCs-CM and CK on Wnt activation, cells were transfected with TOP-flash and TK-RL, 24 hours later, luciferase activity was measured. **B.** Total proteins of QBC939 and Mz-ChA-1 cells were analyzed by western blotting after treated with serum-free medium and MSCs-CM for 24 hours, and **C.** is the statistical results. **D and E.** Expression of cytosolic and nuclear β-catenin in human cholangiocarcinoma cells with MSCs-CM treatment. **F.** Immunofluorescence analysis of β-catenin expression incholangiocarcinoma cells. Scale bar = 20 μm. Abbreviations:MSCs-CM, Mesenchymal stem cell conditioned medium; CK, compound K; MMP2, Matrix Metalloproteinase-2.

So we examined whether MSCs affected Wnt/β-catenin, which promotes the process of cholangiocarcinoma progression, using double fluorescent reporter gene analysis, immunofluorescence staining and western blotting analysis. As shown in Figure 4A, MSCs-CM promotes the activation of Wnt signaling significantly (*p* < 0.01), meanwhile CK inhibited Wnt activation (*p* < 0.05). Western blotting results showed that MSCs-CM significantly up-regulated β-catenin expression, as well as the downstream proteins including *c*-Myc, MMP2 and cyclin D1, compared with control group (*p* < 0.05) (Figure 4B, 4C). β-catenin is a key mediator in Wnt regulating multiple cellular functions. Activation of Wnt signaling leads to cytoplasmic accumulation of β-catenin and allows it translocate into the cell nucleus. We examined the β-catenin expression in cytoplasm and nucleus of QBC939 and Mz-ChA-1 cells by western blotting analysis. Nuclear β-catenin accumulated when treated with MSCs-CM, at the same time, β-catenin expression level was decreased after CK treatment (Figure 4D, 4E). The results of the immunofluorescence staining assay were consistent with western blotting (Figure 4F). These results suggest an important role of MSCs in cholangiocarcinoma cell Wnt/β-catenin activation.

### MSCs promoted cholangiocarcinoma cell invasion through Wnt/β-catenin signaling

**Figure 5:**
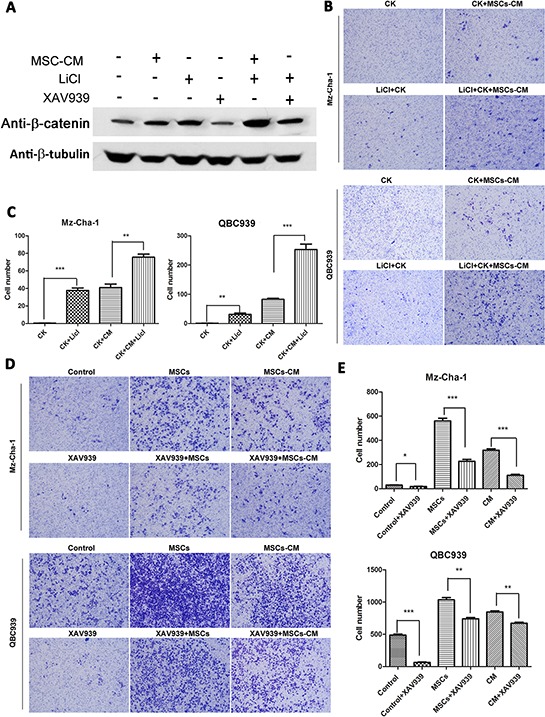
Effects of Wnt/β-catenin signaling on MSCs-mediated cholangiocarcinoma cells invasion **A.** The protein levels of β-catenin in different groups of QBC939 cells (with different treatment: MSC-CM, 20 mM XAV939 or 40 mM LiCl for 24 h) were assayed by western blotting. β-tubulin was used as an internal control. **B–E.** The invasive abilities of the cells were analyzed by an invasion assay using a Matrigel-coated chamber. Different groups of invasive QBC939 and Mz-ChA-1 cells at same time point (24 h after cell seeding) are shown. Data are reported as means ± S.D. of three separate experiments. * and ** indicate *p* < 0.05 and *p* < 0.01, and *** indicates 0.001. Abbreviations: XAV939: an inhibitor of Wnt signaling; LiCl, lithium chloride.

To probe whether the observed MSCs-induced cholangiocarcinoma cell metastasis required Wnt activation, we increased or inhibited β-catenin expression in QBC939 and Mz-ChA-1 cells by LiCl and/or XAV939 (sellckchem, 20 μM) (XAV939, an inhibitor of Wnt/β-catenin signaling), the expression level of β-catenin and cell invasion ability were measured. From Figure 5A, β-catenin expression level was unregulated by MSCs-CM and LiCl, and remarkably inhibited by XAV939. The invasion assay results (Figure 5B, 5C) showed that MSCs and MSCs-CM could promote cell invasion ability. These results underscore the critical importance of the Wnt/β-catenin signaling activation in enabling MSCs to induce metastasis of the cholangiocarcinoma cells.

Taken together, these data suggest the functional effects of hUC-MSCs in the promotion of cholangiocarcinoma cell proliferation, metastatic potency and chemoresistance, both *in vitro* and *in vivo*, and revealed the cellular and molecular mechanisms by which MSCs promote cholangiocarcinoma development. Moreover, we found that metastasis of cholangiocarcinoma is associated with the translocation of β-catenin and upregulation of cyclinD1, *c*-Myc and MMP2. Such findings indicated that MSCs played a promoting role in cholangiocarcinoma cells progression and metastasis.

## DISCUSSION

The discovery that MSCs have the ability to recruit into tumors has led to a great deal of interest over the past decade. Recent studies revealed that MSCs exert multiple effects on tumor development and progression, by increasing stemness of tumor cells, mediating migration, promoting angiogenesis, suppressing immune response and inducing drug resistance. Firstly, we isolated and identified the hUC-MSCs. After that, we confirmed that MSCs could also recruited to cholangiocarcinoma and their metastatic livers in a xenograft tumor model (Figure 2C).

MSCs interact with tumor cells in a myriad of ways, which have the potential to promote or suppress tumor growth and metastasis. Some studies showed that MSCs could promote tumor progression and metastasis [26–28]. While some investigators reported that MSCs could inhibit cancer growth and or inhibit cancer cell metastasis [25, 29, 30]. The study of MSCs on cholangiocarcinoma progression are rarely reported. Liu et al. recent research revealed that hUC-MSCs could inhibit the growth of cholangiocarcinoma xenograft tumors, and conditioned media from hUC-MSCs inhibited proliferation and induced apoptosis of tumor cells in a dose- and time-dependent manner [31]. On the contrary, our study demonstrated a different view of the function of MSCs in cholangiocarcinoma. We observed that both MSCs and MSC-CM promoted the growth of human cholangiocarcinoma cells, and increased the metastasis ability of cancer cells (Figure 2). Multiple *in vitro* assay and *in vivo* models revealed that MSCs and their conditioned medium potently promote QBC939 and Mz-ChA-1 cells growth and increase subcutaneous tumor growth (Figure 1). These results may be due to different cell lines, tumor models, times and dose of MSCs were used, culture methods, or other unknown factors. As we know, MSCs could play a dual role on many cancer progression. The soluble factors may be the key factor to lead these difference. Thus, a thorough characterization of the MSCs secreted cytokines in the tumor microenvironment should be a important focus in future research.

Cholangiocarcinoma is an aggressive malignancy originating from the bile duct epithelium. It is recognized as a highly metastatic cancer. Cholangiocarcinoma patients are mostly clinically silent and difficult to diagnose until the metastatic stage, leading to a poor prognosis. A better understanding of the molecular pathogenesis of the metastasis of cholangiocarcinoma is needed. Epithelial-Mesenchymal Transition (*EMT*) is believed to be a major mechanism by which cancer cells become migratory and invasive [32]. Techasen et al. reported that E-cadherin can act as a central modulator of tumor cell phenotype and is a potential metastasis marker in cholangiocarcinoma [33]. In this study, we also tested the EMT markers by Real-time PCR, but we did not find the Epithelial-to-Mesenchymal Transition when cholangiocarcinoma cell lines treated with MSC-CM (Supplementary Figure S3). The Wnt/β-catenin signaling pathway plays a crucial role in the regulation, differentiation, proliferation and cellular death processes [34]. Abnormal regulation of Wnt/β-catenin signaling is linked to a variety of human diseases, such as malignancy [35]. Then, we measured β-catenin expression in 42 cholangiocarcinoma samples, and investigated the correlation between the expression pattern and clinic pathologic factors (Supplementary Figure S2, Table 1). Our results revealed that β-catenin was related to PTNM of cholangiocarcinoma (Table 2), β-catenin and its target gene *c*-myc expression level are higher in PTNM III than other samples (Table 3). According to Keishi Sugimachi et al, no β-catenin mutations have been reported for intrahepatic cholangiocarcinoma, and reduced membranous expression and nuclear translocation of β-catenin are involved in cholangiocarcinogenesis, progression and invasion [36]. In addition, we investigated on β-catenin and β-catenin-related molecules, including MMP-2, *c*-Myc, in human cholangiocarcinoma cell lines QBC939 and Mz-ChA-1 when treated with MSCs or MSC-CM. We had observed that β-catenin expression level was up-regulated and translocated to nucleus (Figure 4). These results suggest that MSCs may promote cholangiocarcinoma cell metastasis through Wnt/β-catenin pathway.

**Table 3:** *c*-Myc expression in different cholangiocarcinoma tissues

pathological factors	numbers	*c*-Myc		positive (%)	χ2	*p*
		−	+			
**sex**						
male	21	13	8	38.1	0.0988	>0.05
female	21	12	9	42.9		
**age**						
≤60	30	17	13	43.3	0.2711	>0.05
> 60	12	8	4	33.3		
**PTNM**						
I	3	1	2	66.7	0.9198	>0.05
II + III	39	24	15	38.5		
**Infiltration depth**						
T1+T2	22	13	9	40.9	0.0036	>0.05
T3+T4	20	12	8	37.5		
N_0_	32	5	12	37.5	7.435	<0.05
N_1_	10	12	5	50.0		

Currently, the standard chemotherapy for cholangiocarcinoma includes oxaliplatin or cisplatin [37]. But the tumors are resistant to these conventional chemotherapy [38]. Inadequate response to therapy is more likely as a result of the molecular and cellular heterogeneity of the primary tumor, which moreover may be linked to both malignant and recurrent disease. The mechanism caused cancer cell drug resistance include promoting cell proliferation, loss of tumor suppressor gene function, inactivation of cell death or enhancement of survival functions and activation of telomerase. There is another mechanism, called environment mediated-drug resistance (EMDR), also played a vital role in drug resistance. EMDR contains soluble factor-mediated drug resistance and cell adhesion-mediated drug resistance [16]. We exposed QBC939 and Mz-ChA-1 with CK in presence or absence of MSC-CM, and then analyzed the cell viability and the invasion ability. The results showed that the proliferation and invasion ability were increased compared with the CK treated group (Figures 2A, 3A). That means the anti-tumor effect of CK was decreased at the presence of MSCs-CM. From *in vivo* study, on the condition of MSCs, the tumor inhibition rate of CK was also decreased. These results showed that MSCs promote the chemoresistance of cholangiocarcinoma, and suggested that soluble factors, such as cytokines, chemokines and growth factors secreted, may be the main aspect inducing the drug resistance.

In conclusion, our present studies demonstrated that MSCs and MSC-CM could both significantly promote the proliferation and increased the metastasis in human cholangiocarcinoma cells through Wnt/β-catenin pathway. Additionally, MSCs may also be involved in the chemoresistance of cholangiocarcinoma cell. The elucidation of the mechanism of MSCs promoting tumor cell growth and metastasis provides evidence of MSCs on cancer progression. The tumor-promoting molecules secreted by MSCs or the pathway activated by MSCs in tumor cells needs to be studied deeply, which may enrich the list of potential targets for molecular therapy.

## MATERIALS AND METHODS

### Mice and ethics statement

Male BALB/c-nu/nu mice, at age of 3–5 weeks, were purchased from Xiamen University Laboratory Animal Center. All experimental mice were maintained under SPF conditions and raised under standard conditions (12-hour day-night rhythm). All animal procedures were approved by the Animal Care and Use Committee of Xiamen University (license No: SYXK [Min] 2008–0003, issued on May 6, 2008).

### Cell lines

Human umbilical cord blood samples were obtained from the umbilical vein immediately after delivery with the informed consent of the mother. Sample collection was approved by the Xiamen Zhongshan Hospital. hUC-MSCs were obtained and characterized as described previously [39]. MSCs were cultured with MSC medium (STEMCELL Technologies, Vancouver, CA) supplemented with 10% fetal bovine serum (FBS) (Gibco, Grand Island, NY) and 100 U/mL penicillin/streptomycin. The third to eighth passages of MSCs were used in the following experiments. Human cholangiocarcinoma cell lines QBC939 and Mz-ChA-1(kindly provided by the First affiliated hospital of Xiamen university) were cultured in RPMI 1640 (Gibco, Grand Island, NY) supplemented with 10% FBS and 100 U/mL penicillin/streptomycin solution. All cells were maintained in a humidified 5% CO_2_ environment at 37°C.

### Identification of MSCs

MSCs were isolated from human umbilical cord and characterized by flow cytometric analyses with CD29, CD34, CD44, CD45 and CD90 antibodies. Cells were cultured and harvested after they grow to 80% of the dish. After been washed with phosphate buffered saline (PBS) twice, MSCs were stained with antibodies against CD29, CD34, CD44, CD45 and CD90, and IgG1 or IgG2b added as the isotype. The samples were analyzed by Flow cytometry (Becton Dickinson, Franklin Lakes, NJ, USA). For adipogenic and Osteogenic differentiation assay, Human Umbilical Cord MSC Adipogenic Differentiation Medium and Osteogenic differentiation Medium (cyagen, Guangzhou, CHN) were used according to the protocol.

### Preparation of MSCs conditioned media (MSC-CM)

hUC-MSCs were cultured to 90% confluence. Washed with PBS twice then add serum-free RPMI-1640 to each dish for 24 hours. The conditioned media were filtered through the 0.22 mm pore sterile filter and stored at −80°C until further use within one week.

### Apoptosis and cell proliferation assay

Cholangiocarcinoma cells were cultured and treated with different concentration of CK. After 24 hours, cells were trypsinized and harvested. Centrifuged and washed twice, the cells were resuspended and stained for annexin V and propidium iodide (PI) as described in the manufacturer's instructions (Pharmingen, San Diego, CA, USA). The samples were analyzed by Flow cytometry (Becton Dickinson, Franklin Lakes, NJ, USA). For proliferation assay, cholangiocarcinoma cells were cultured in 96 well dished and cell proliferation assay assessed by methyl thiazolyl tetrazolium (MTS) assay (promega, Madison, WI, USA).

### EdU proliferation assay

QBC939 and Mz-chA-1 cells were seeded in 96-well plate. The cells were incubate under each conditions for 24 hours. Cell proliferation was detected using the incorporation of 5-ethynyl-29-deoxyuridine (EdU) with the EdU Cell Proliferation Assay Kit (Ruibo, Guangzhou, China). The number of cells that incorporated EdU was determined by fluorescence microscopy.

### Colony-forming assay

Cells were seeded in six-well plates at a density of 500 cells/well and maintained in complete medium overnight. Then treated with MSC-CM, CK for 12 hours. Cells were washed with PBS and cultured in complete medium for about 2 weeks, after most of the colonies had expanded to more than 50 cells, the cells were washed with PBS, fixed in 4% paraformaldehyde for 15 min and stained with crystal violet for 10 min. The colonies were counted. Three independent experiments were carried out for each assay.

### Transwell assay

60 μl serum-free diluted Matrigel (0.8 mg/mL) was added into the upper chamber (Corning, MA, USA) using cooled pipet tips. Incubate the plates at 37°C for 2 hours before starting the invasion assay. The cholangiocarcinoma cells (1 × 10^6^ cells) were added into the upper chamber and added FBS-free medium, MSCs, MSC- CM, CK+MSC-CM and CK into the lower chamber. 48 hours later, remove the cell which were not migrated to the the reverse side of the filters and washed with PBS. Then 4% paraformaldehyde fixed the migration cells that had invaded across the Matrigel and passed through the transwell filter for 10 min and stained with Giemsa stain and counted using bright-field microscopy (IX51, Olympus Corporation, JPN).

### Dual-Glo™ luciferase assay

We use the TOPFLASH firefly luciferase and TK-RL Renilla luciferase constructs to measure the activation of the Wnt pathway after MSC-CM and CK treatment. Cells grown on 24-well plates were transfected in quadruplicates with cDNAs (20 ng/well) for TCF-luciferase reporter (TOPflash) along with a control Renilla plasmid (TK-RL) by Lipofectamine 2000 transfection Reagent (Invitrogen, Carlsbad, CA, USA). Twenty-four hours post-transfection, the cells were treated with MSC-CM or CK, 24 hours later the cells were lysed and the luciferase activity was measured and normalized to the corresponding Renilla activity using the dual-luciferase assay kit (Promega, Madison, WI, USA).

### Western blotting

Samples were collected by lysing cells in RIPA lysis buffer (50 mM Tris, pH 7.4, 150 mM NaCl, 1 mM ethylenediaminetetraacetic acid (EDTA), 0.1% SDS, 1% TritonX-100, 1% sodium deoxycholate, and 1 mM phenylmethylsulfonyl fluoride (PMSF). Each sample was size-fractionated using SDS-polyacrylamide gel electrophoresis (PAGE) and electrotransferred onto polyvinylidene difluoride (PVDF) transfer membranes (Dupont, Boston, MA, U.S.A.). Blots were incubated for 1 h at room temperature in 5% skim milk for blocking, and proteins were detected with primary antibodies overnight at 4°C, and then blotted with horseradish peroxidase conjugated secondary antibodies for 1 hour at room temperature. The immunoblots were visualized using ECL (GE Healthcare, Bucks, UK).

### Xenograft assays in nude mice

For xenograft experiments, QBC939 cells were implanted alone (2 × 10^6^/mice), or were mixed with MSCs (3:1) [25] by subcutaneous injection into the right foreleg of the mouse. Mice in control groups were given QBC939 or MSCs alone. Then 10 mg/kg CK or 200 μl MSC-CM was administered to the mice by intraperitoneal injection every other day for 30 days. Nude mice were used at 4–6 weeks of age. Tumor volume was calculated as 1/2ab^2^.

### Immunohistochemistry

Tumor tissue specimens were fixed in neutral formalin and embedded in paraffin after collection from the sacrificed mice. Tissue sections were cut and dewaxed, then incubated with 0.01 M natrium citricum for antigen retrieval. The slides were rinsed in phosphate-buffered saline and incubated overnight at 4°C with diluted anti-cyclinD1, anti-*c*-Myc, anti-ki67 or anti-β-catenin antibodies. Following steps were performed using the immunostaining kit (Maixin BIO, Fuzhou, China) according to the manufacturer's instructions.

### Immunofluorescence staining

Immunofluorescence staining was performed as previously [40]. Briefly, after CK or MSC-CM treatment, cells were fixed with 4% paraformaldehyde and permeabilized with 0.1% Triton X-100. Then, cells were incubated with first antibodies (1:200) overnight at 4°C. The cells were then washed and subsequently incubated with Cy3-conjugated secondary antibodies at a dilution of 1:200 for 1 h at room temperature. After that, cells were washed and DAPI was stained for 5 min at room temperature. At last, cells were mounted in ProLong antifade solution onto glass slides and observed under multi-photon laser scanning microscope (FV1000, Olympus Corporation, JPN).

### *In vivo* imaging of homing ability to tumors

We use Cell Tracer CM-Dill (Invitrogen Life Technologies, CA, USA) to trace MSCs *in vivo*. CM-Dil working solution was prepared as the manufacturer's instructions. Briefly, 1 mg CM-Dil/ml stock solution in culture-grade DMSO, 8 μM solutions were made in 500 μl PBS, vortexed, and then combined with 2 × 10^6^ hUC-MSCs in 500 μl PBS, to give 10^6^ cells/ml in 4 μM CM-Dil labeling solution. CM-Dil cell suspensions were incubated for 30 min at 37°C and then for 15 min at 4°C. After labeling, cells were washed three times with PBS and resuspended in fresh medium. 24 hours after staining, cells were injected into the cauda vein (10^6^/cells) of tumor-bearing mice or mixed MSCs tumor bearing mice, and then optical bioluminescence imaging was conducted to periodically trace the cells using a maestro *in vivo* imaging system (CRI, MA, USA)

### Real-time qPCR

Total cellular RNA was prepared using TRNzol reagent (Tiangen, Beijing, CHN) and the expression levels of *E-cadherin*, *Vimentin*, *N-cadherin*, *Snai1*, *Slug*, *ZEB1* and *ZEB2* mRNAs were determined by real-time reverse transcriptase–PCR using GoTaq^®^ qPCR Master Mix (promega, Madison, WI, USA). Data shown are normalized to GAPDH expression and represent the average of three repeated experiments. The primers for specific genes were shown in Table S1.

### Statistical analysis

Data are presented as the means ± S.D. for at least three separate determinations for each group. The differences between the groups were examined for statistical significance using the Student's *t*-test with SPSS software. Differences were considered significant when the *p* < 0.05.

## SUPPLEMENTARY FIGURES AND TABLE


