# Supplementary material for: Involvement of Wnt/β-catenin signaling in the mesenchymal stem cells promote metastatic growth and chemoresistance of cholangiocarcinoma
Source: Oncotarget. 2015 Oct 14;6(39):42276–89. doi: 10.18632/oncotarget.5514 (PMC4747224; doi:10.18632/oncotarget.5514)
Supplement: Supplementary file 1 [file oncotarget-06-42276-s001.pdf]

SUPPLEMENTARY FIGURES AND TABLE

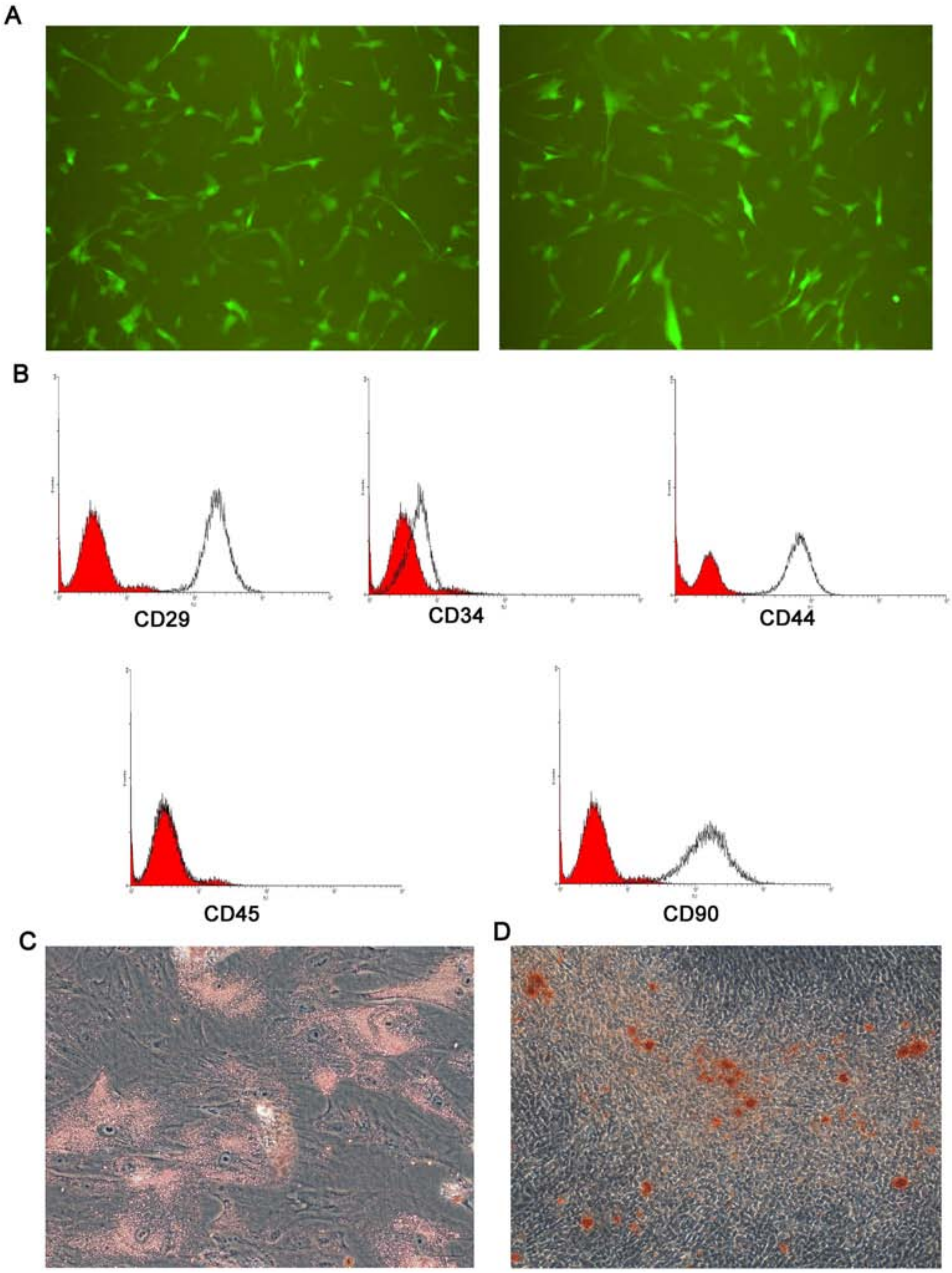

Supplementary Figure S1: Characterization and of hUC-MSCs. A. Morphology of MSCs-GFP. **B.** Human umbilical cord-derived MSCs were characterized by flow cytometry assay with CD29, CD34, CD44, CD45 and CD90 antibodies, and the IgG1 and IgG2b are the isotype. **C.** Oil Red O staining of adipogenic differentiated hUC-MSCs (200×). **D.** Alizarin Red S staining of osteogenic differentiated hUC-MSCs (200×).

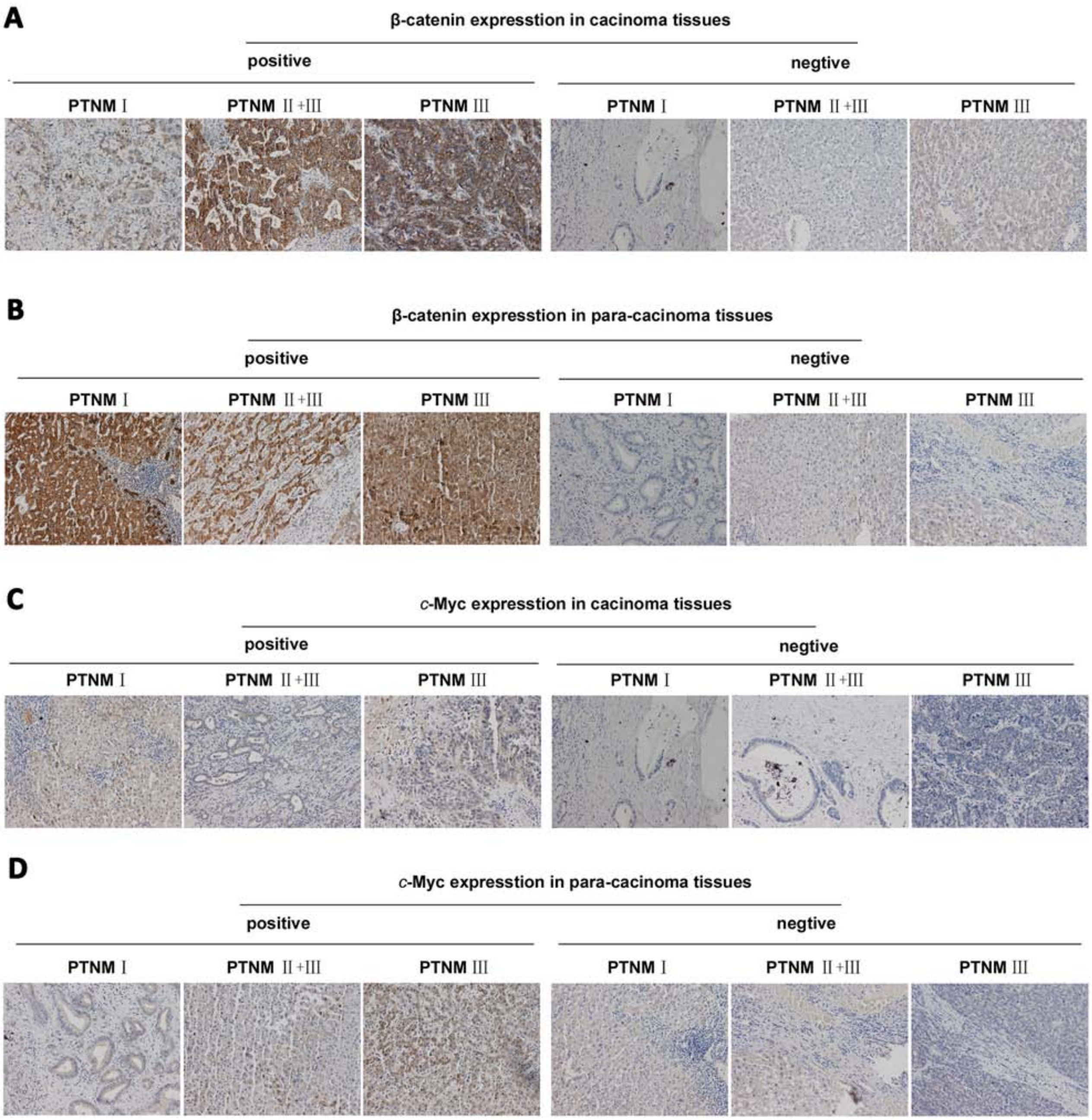

Supplementary Figure S2: Immunohistochemical staining of β-catenin and c-myc in cholangiocarcinoma and para-carcinoma tissues. Representative images of antibody staining in cholangiocarcinoma and para-carcinoma; **A and B.** β-catenin +positive and –negative, **C and D.** c-myc +positive and –negative.

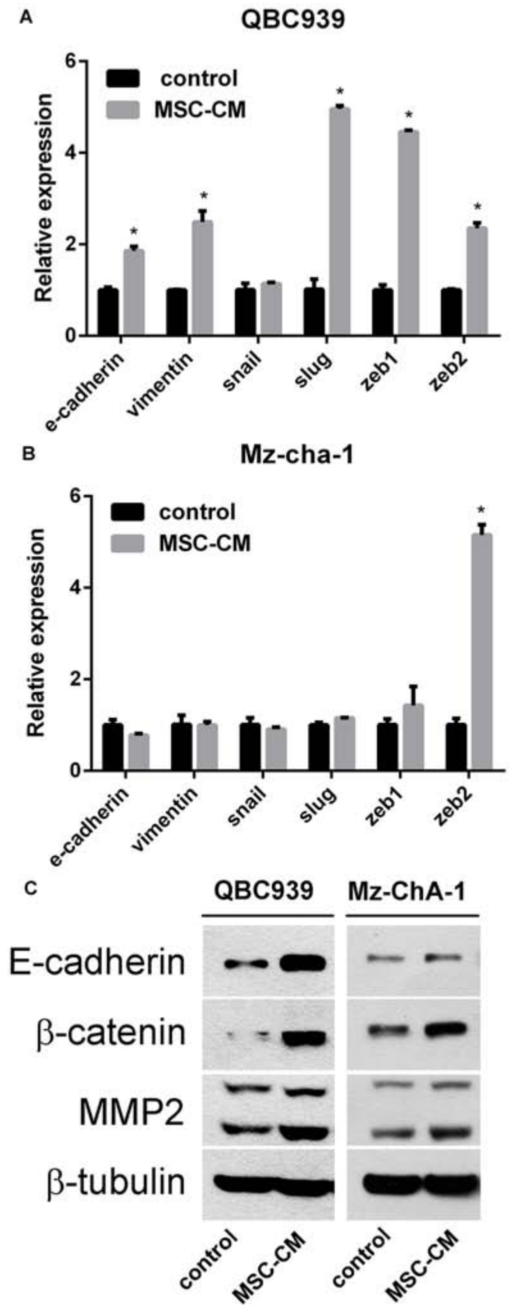

Supplementary Figure S3: Effects of MSC-CM on cholangiocarcinoma cells epithelial-mesenchymal transition (EMT). A and B. Relative levels of mRNAs encoding *E-cadherin, vimentin, Snail, Slug, ZEB1* and *ZEB2* in QBC939 and Mz-ChA-1 cells were determined by real-time qPCR. *GAPDH* mRNA was used to normalize variability in template loading. Error bars represent the mean ± S.D. of triplicate experiments. * indicate $p < 0.05$. (B) Western blot analysis was used to evaluate E-cadherin in cell lysates. After QBC939 and Mz-ChA-1 cells treated with MSC-CM for 3 days, the expression of the β-catenin, MMP2, E-cadherin were increased. Abbreviations: EMT, epithelial-mesenchymal transition; GAPDH, glyceraldehyde-3-phosphate dehydrogenase; ZEB1, zinc-finger E-box binding homeobox 1; ZEB2, zinc-finger E-box binding homeobox 2; MMP2, Matrix Metalloproteinase-2.

Supplementary Table S1: Real-time RT-PCR Primers

Gene	Forward primer(5′-3′)	Reverse primer(5′-3′)
E-cadherin	TGCCCAGAAAATGAAAAAGG	GTGTATGTGGCAATGCGTTC
Vimentin	GAGAACTTTGCCGTTGAAGC	GCTTCCTGTAGGTGGCAATC
Snail	CTGGGTGCCCTCAAGATGCA	CCGGACATGGCCTTGTAGCA
Slug	TACCGCTGCTCCATTCCACG	CATGGGGGTCTGAAAGCTTGG
ZEB1	TGCACTGAGTGTGGAAAAGC	TGGTGATGCTGAAAGAGACG
ZEB2	CGGTATTGCCAACCCTCTGGA	TTGTTGTGCCAGGGGTGTTCC
GAPDH	TGCACCACCAACTGCTTAGC	GGCATGGACTGTGGTCATGAG
